# Germinal matrix hemorrhage-intraventricular hemorrhage: pathogenesis and outcomes

**DOI:** 10.1186/1824-7288-41-S1-A31

**Published:** 2015-09-24

**Authors:** Ettore Piro

**Affiliations:** 1 DepartmentofSciencesforHealth Promotion andMother-ChildCare “G. D'Alessandro”, University of Palermo, Italy

## 

Germinal matrix hemorrhage-intraventricular hemorrhage (GMH-IVH) is one of the CNS injuries affecting preterm infants occurring in about 15%–20% of subjects weighing less than 1500 g. Currently, using ultrasonography, we recognize three grades of GMH-IVH. Grade I involving the subependymal parenchyma and/or extending in less than 10% of the ventricle, grade II with intraventricular bleeding not expanding in more than 50% of the ventricle, grade III characterized by consistent (> 50%)intraventricular bleeding with ventricular dilatation. A concomitant intraparenchymal lesion (IPL), due to a venous infarction (ex grade IV), can be associated with any grade of IVH, worsening the prognosis.

Pathogenesis of GMH-IVH is multifactorial and complex, due to several factors identified as genetic predisposition, systemic (cardiorespiratory, hematologic, immunologic and metabolic) and developmental (immature anatomical substrates and immature, impaired cerebrovascular reactivity of the preterm brain) predisposing to intraventricular bleeding. Prenatal chorioamnionitis with umbilical vasculitis, primarily due to Ureaplasma species, is considered the main prenatal factor responsible for increased risk of GMH-IVH [1,3].

Of primary importance for the occurrence of intraventricular bleeding is considered the hemodynamic instability that, in the first three days of life, affects the extreme preterm, in which cerebral vasoreactivity and autoregolatory mechanisms to pressure variability are weak. In extremely preterm newborns, hypercapnia (paCo2 >55 mmHg), with consequent cerebral vasodilatation, arterial hypotension and persistent patent ductus arteriosus with diastolic steal, are responsible for the cerebral blood flow instability. Neonatal intubation, ventilatory strategies, intravenous fluid and electrolytes management and neonatal complications also play an important role.

In about 35% of infants with intraventricular bleeding a posthemorragic ventricular dilatation (PHVD) will occur, with possible evolution in about 22% in posthemorrhagic hydrocephalus (PHH), that in about 9% will require the placement of a permanent shunt, therefore complicating the outcomes [2]. Of primary importance is the treatment of symptomatic PHH. Communicanting and non communicanting hydrocephalus have different treatment options. Some preterm infants may be affected by a transient symptomatic form, and therefore need a short period of CSF diversion. In communicating hydrocephalus serial lumbar puncture, better if performed as “early intervention”, are associated with a reduced need of persistent shunt insertion. Ventriculoperitoneal shunt is the more frequent form of permanent CSF diversion used in PHH. Infections and shunt revisions are its main complications. In non communicating hydrocephalus endoscopic third ventriculostomy is an important alternative [3]. Survivors can be affected by neurological, neurosensorial, cognitive and behavioral impairment depending from the individual risk profile.
